# A New Specimen of Carroll’s Mystery Hupehsuchian from the Lower Triassic of China

**DOI:** 10.1371/journal.pone.0126024

**Published:** 2015-05-27

**Authors:** Xiao-hong Chen, Ryosuke Motani, Long Cheng, Da-yong Jiang, Olivier Rieppel

**Affiliations:** 1 Wuhan Centre of China Geological Survey, Wuhan, Hubei, P. R. China; 2 Department of Earth and Planetary Sciences, University of California Davis, Davis, California, United States of America; 3 Laboratory of Orogenic Belt and Crustal Evolution, MOE, Department of Geology and Geological Museum, Peking University, Beijing, P. R. China; 4 Center of Integrative Research, The Field Museum, Chicago, Illinois, United States of America; University of Naples, ITALY

## Abstract

A new specimen of an enigmatic hupehsuchian genus is reported. The genus was first recognized by Robert L. Carroll and Zhi-ming Dong in 1991, who refrained from naming it because of the poor quality of the only specimen known at the time. After more than two decades, we finally report a second specimen of this genus, which remained unprepared until recently. The new specimen preserves most of the skeleton except the skull, allowing us to erect a new genus and species, *Eretmorhipis carrolldongi*. The new species shares many characters with *Parahupehsuchus longus*, including the strange axial skeleton that forms a bony body tube. However, the body tube is short in the new species, being limited to the pectoral region. The vertebral count and limb morphology considerably differ between the new species and *P*. *longus*. The forelimb of *E*. *carrolldongi* is markedly larger than its hind limb as in *Hupehsuchus nanchangensis* but unlike in *P*. *longus*. The new species is unique among hupehsuchians in a list of features. It has manual and pedal digits that spread radially, forming manus and pes that are almost as wide as long. The third-layer elements of the dermal armor are unusually large, spanning four vertebral segments, yet there are substantial gaps among them. With the addition of the unique paddle, it is now clear that Hupehsuchia had diverse forelimb morphologies spanning from paddles to flippers, unlike ichthyopterygians that were taxonomically more diverse yet only had flippers.

## Introduction

Hupehsuchia is a peculiar clade of Early Triassic marine reptiles that is known exclusively from Hubei Province, China. Within Hubei Province, only two counties are known to yield these reptiles, namely Nanzhang County of Xiangyang City and Yuan’an County of Yichang City, located in the mid-northern part of the Province. The two counties lie next to each other, although they belong to different cities. Four monotypic genera have been named within this group, namely *Nanchangosaurus suni* [1], *Hupehsuchus nanchangensis* [2], *Parahupehsuchus longus* [3], and *Eohupehsuchus brevicollis* [4].

In 1991, Robert L. Carroll and Zhi-ming Dong published a landmark paper on hupehsuchian paleontology that reviewed the specimens known at the time [2]. Among the many contributions of the paper was the first suggestion that *Nanchangosaurus* and *Hupehsuchus* formed a group—until then, *Nanchangosaurus* was considered to be a sauropterygian [1, 5], and *Hupehsuchus* a possible ‘thecodontian’ [6]. The grouping of the two genera was later confirmed by phylogenetic analyses [7], which also indicated that hupehsuchians were not sauropterygians or thecodontians but the sister group of Ichthyosauriformes.

In the same paper of 1991 [2], a third form of Hupehsuchia was recognized and a suggestion was made that it merited a separate generic status—note that it was called a second genus rather than a third in the paper, which placed *Nanchangosaurus* in the third place, and that *Parahupehsuchus longus* and *Eohupehsuchus brevicollis* were not yet known at the time. The authors observed many differences in the limb skeletons between this form and *Hupehsuchus nanchangensis*, while noting the overall resemblance between the two in terms of skeletal proportions. They also pointed out that the neural spines of the third form were proportionately shorter than in *H*. *nanchangensis* relative to the body. Despite these differences, the authors refrained from naming a new genus and species because they recognized that the only specimen, IVPP V4070 (Institute of Vertebrate Paleontology and Paleoanthropology, Academia Sinica), would not warrant a status as the holotype. Although the specimen represented a large part of a skeleton excluding the skull and the tip of the tail, most bones were only known as natural impressions that are not necessarily well defined. The genus remained unnamed until today, for about 23 years.

In 2003, a hupehsuchian specimen that resembled this enigmatic genus was reported [8]. The specimen represents an almost complete individual but it was not described in detail because the purpose of the paper was to report the polydactyly seen in the specimen. The specimen remained unnamed, and has not been re-studied since.

Wuhan Centre of China Geological Survey (WGSC) has been undertaking an ongoing fieldwork program in Nanzhang and Yuan’an Counties since 2011. It has yielded many specimens that contributed new pieces of information on hupehsuchian paleontology [3, 4, 7]. This research activity led to preparation of a fossil specimen that was donated to WGSC in the previous century, showing that it resembled IVPP V4070. The specimen finally reveals the detailed postcranial anatomy of the mystery genus recognized by [2] in 1991. The purpose of the present paper is to report the new specimen and compare it to other hupehsuchian specimens, including IVPP V4070.

## Materials and Methods

### Specimens

The main specimens for the present study are WGSC V26020 and IVPP V4070. WGSC V26020 was donated by People’s government of Yuan’an County in 1970s, and is now accessioned to the fossil collection at the central facility of WGSC, located in Wuhan, Hubei Province, China. The specimen was collected through a field excavation in Yuan’an County, Hubei Province, China by the local government. IVPP V4070 is a specimen previously studied by [2]. It is accessioned at the main facility of IVPP in Beijing, China. We also included a hupehsuchian specimen studied by [8] for comparison based on literature description.

### Phylogenetic Analysis

Our analysis is based on a modified version of a published data matrix [4], which already contained IVPP V4070. We added two specimens, namely WGSC V26020 and the specimen of [8], as well as five new characters to account for the expanded taxonomic scope. There are a total of 37 discrete characters, all of which are binary. We treated the new taxon at specimen level to test if they indeed group together under the parsimony criterion. The data matrix is available in S1 File.

We analyzed the resulting data matrix using PAUP* 4b10 (Phylogenetic Analysis Using Parsimony) [9]. A branch and bound search, which ensures discovery of all most parsimonious trees, was used given the small data matrix. The result was confirmed by an ienum search in TNT 1.1 [10], which was also used to calculate the Bremer index and bootstrap (n = 1000) values.

### Nomenclatural Acts

The electronic edition of this article conforms to the requirements of the amended International Code of Zoological Nomenclature, and hence the new names contained herein are available under that Code from the electronic edition of this article. This published work and the nomenclatural acts it contains have been registered in ZooBank, the online registration system for the ICZN. The ZooBank LSIDs (Life Science Identifiers) can be resolved and the associated information viewed through any standard web browser by appending the LSID to the prefix “http://zoobank.org/”. The LSID for this publication is: urn:lsid:zoobank.org:pub:132627FE-9142-4EE2-BACB-08CAFC65C3E2. The electronic edition of this work was published in a journal with an ISSN, and has been archived and is available from the following digital repositories: PubMed Central, LOCKSS.

## Results

### Phylogenetic Analysis

**Fig 1:**
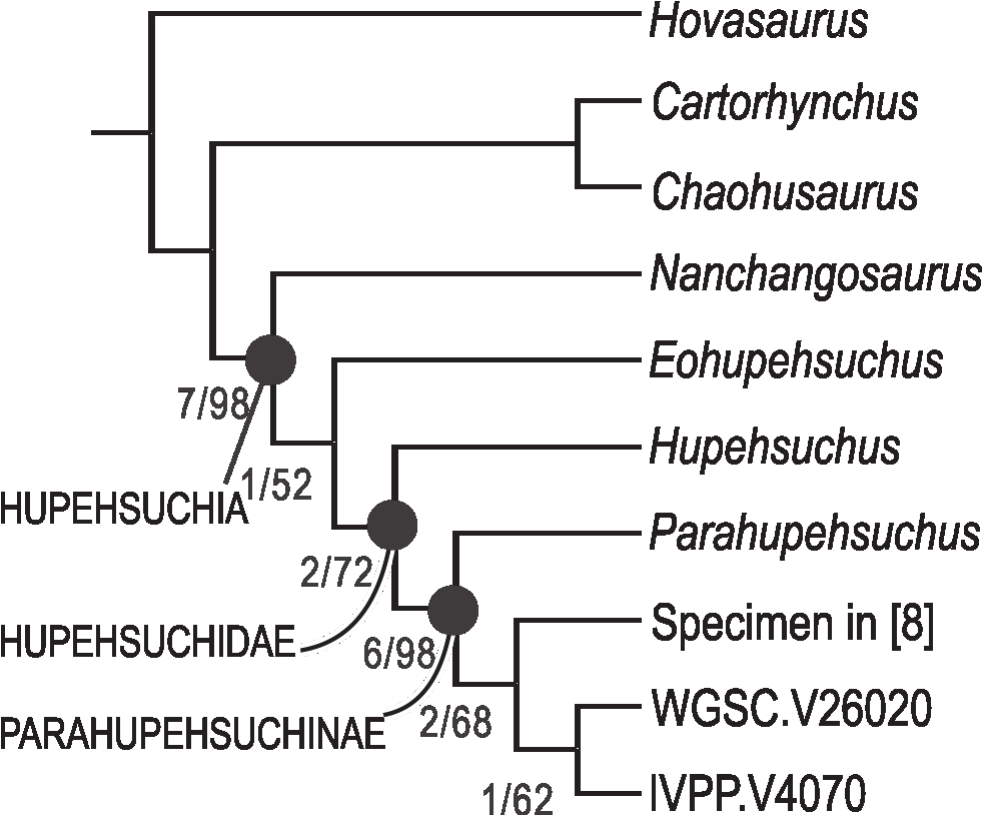
Phylogenetic hypothesis of Hupehsuchia. WGSC.V26020 and IVPP.V4070 represent *Eretmorhipis carrolldongi* gen. et sp. nov. The number associated with nodes are the Bremer index/bootstrap (n = 1000) values. See text for details.

A branch and bound search in PAUP* 4b10 found a single most parsimonious tree (TL = 50, CI = 0.74, RI = 0.806), as depicted in Fig 1. The same topology was found by TNT 1.1. The new specimen formed a clade with IVPP V4070, supporting the view that the two are conspecific. The specimen described by [8] became the sister taxon of the clade formed by WGSC V26020 and IVPP V4070. See Fig 1 for the Bremer index and bootstrap values.

### Systematic Paleontology

#### Systematic hierarchy

Reptilia Laurenti 1768 [11]

Diapsida Osborn 1903 [12]

Hupehsuchia Young 1972 [6]

Hupehsuchidae Young 1972 [6]

#### Revised Diagnosis

Ribs pachyostotic mid-shaft; longitudinal groove on ribs absent; at least some dorsal ribs articulating with two vertebrae; dorsal vertebral count at least 28; dermal armor third layer elements up to two vertebrae long.

Parahupehsuchinae nov.

#### Definition

All hupehsuchids that are more closely related to *Parahupehsuchus longus* than to *Hupehsuchus nanchangensis*.

#### Diagnosis

Craniad dorsal rib with extensive posterior and anterior flanges that overlap adjacent rib; 30 or more dorsal vertebrae; dermal armor above anterior caudal vertebrae; fifth metacarpal robust; extra proximal carpal and tarsal.

*Eretmorhipis carrolldongi* gen. et sp. nov urn:lsid:zoobank.org:act:23308E45-3021-483D-AB7F-10496DE49E3B

#### Etymology

Generic name combines ερετμον (Gr. oar) with ῥιπίς (Gr. fan), referring to the fan-shaped paddle typical of the new genus. The specific name acknowledges the contribution of Robert L. Carroll and Zhi-ming Dong, who first recognized the distinctiveness of the species based on IVPP V4070 [2].

#### Holotype

**Fig 2:**
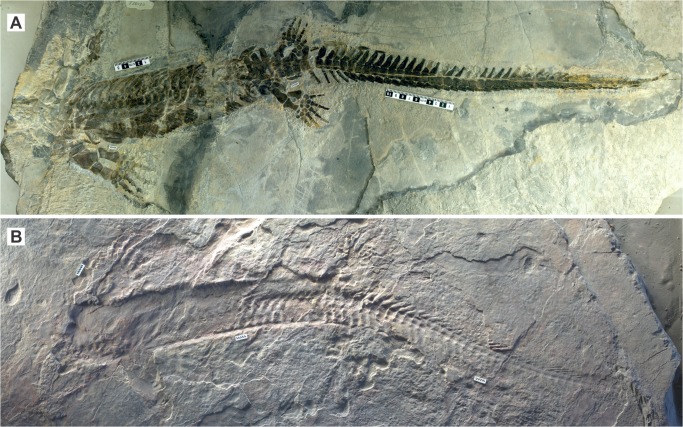
Whole views of the two specimens of *Eretmorhipis carrolldongi* gen. et sp. nov. **A, the holotype, WGSC V26020.** B, referred specimen, IVPP V4070 (the original specimen of [2]). Note that IVPP V4070 mostly comprise skeletal impressions. Scale bars are in centimeters.

**Fig 3:**
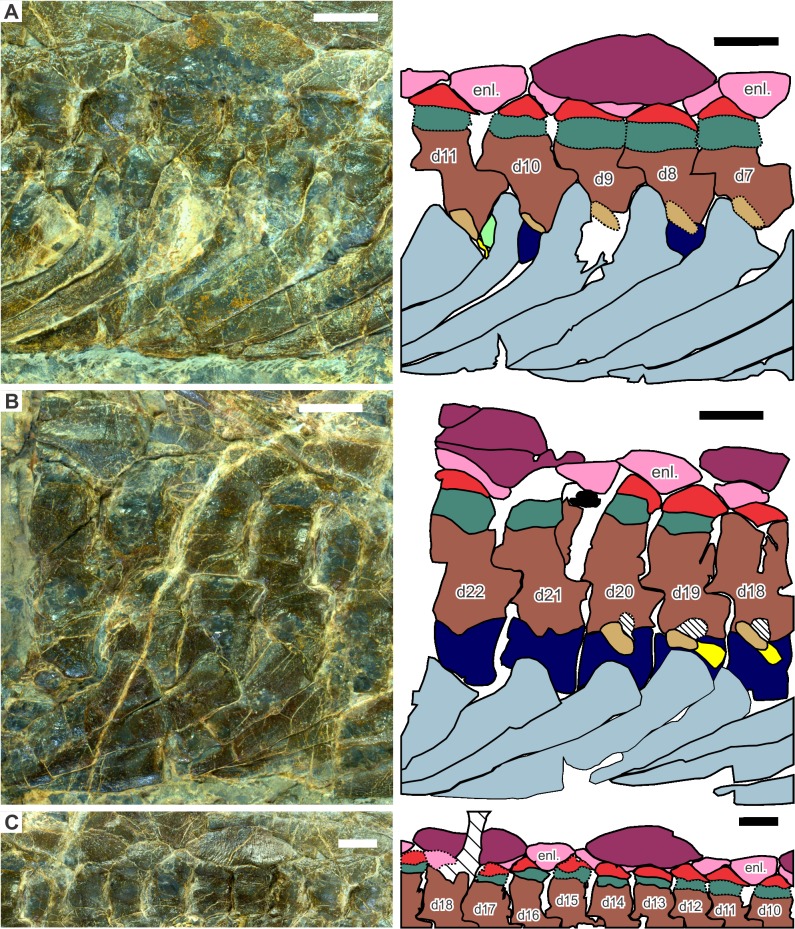
Dorsal axial skeleton of *Eretmorhipis carrolldongi* gen. et sp. nov, as seen in the holotype (WGSC V26020). A, anterior dorsal region; B, posterior dorsal region; C, pattern of dermal ossicle distribution. Colors: black, unidentified elements; brown, neural arch and first neural spine segment; dark blue, vertebral centra; green, second neural spine segment; light blue, rib; light brown, diapophysis; light green, limb elements; light yellow, girdle elements; mint green, dorso-craniad extension of parapophysis; orange, lateral gastral elements; pink, second layer of dermal armor; purple, hemal arch and spine; red, first layer of dermal armor; red-purple, third layer of dermal armor; white, median gastral elements; yellow, parapophysis. Vertebral position is indicated by numbers starting with d, indicating ‘dorsal’, while enl. denotes enlarged second layer dermal armor elements. Hatched areas are damaged. Scales are 1 cm long.

**Fig 4:**
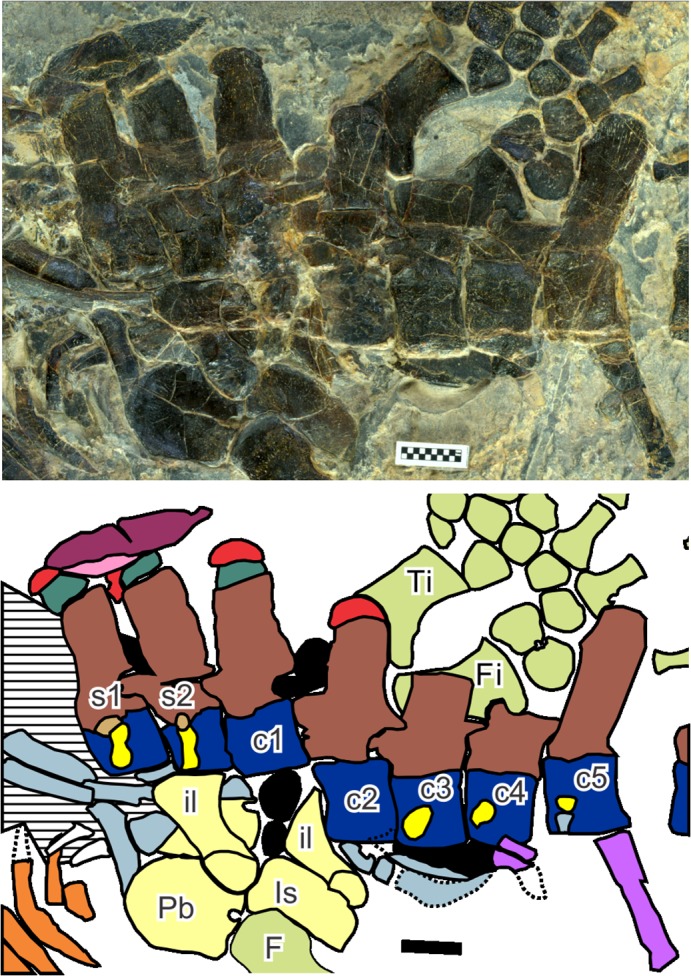
Pelvic region of *Eretmorhipis carrolldongi* gen. et sp. nov, as seen in the holotype (WGSC V26020). Symbols: F, femur; Fi, fibula; il, ilium; Is, ischium; Pb, pubis; Ti, tibia. Numbers starting with s indicate sacral vertebral positions, while those with c are caudal vertebral positions. See Fig 3 for colors. Area filled with horizontal lines contains many identified bones that are damaged. Scales are 1 cm long.

**Fig 5:**
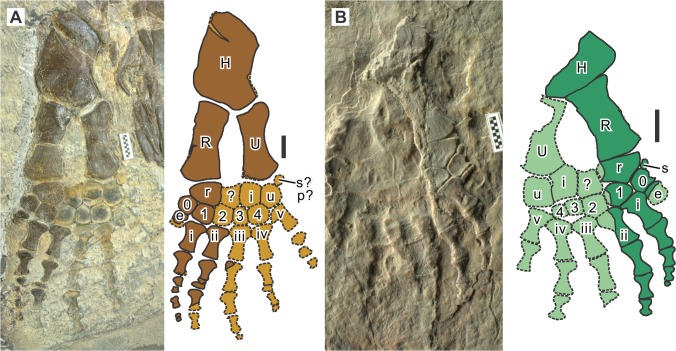
Forelimb of *Eretmorhipis carrolldongi* gen. et sp. nov. **A, WGSC V26020; B, IVPP V4070.** Symbols: e, extra preaxial metacarpal; H, humerus; i, intermedium; p, pisiform; R, radius; r, radiale; s, sesamoid; U, ulna; u, ulnare, 0–4, distal carpal; i-v, metacarpal. Brown indicates the left limb and green the right limb. Lighter colors with broken outline are impressions. Scale bars are 1 cm long.

**Fig 6:**
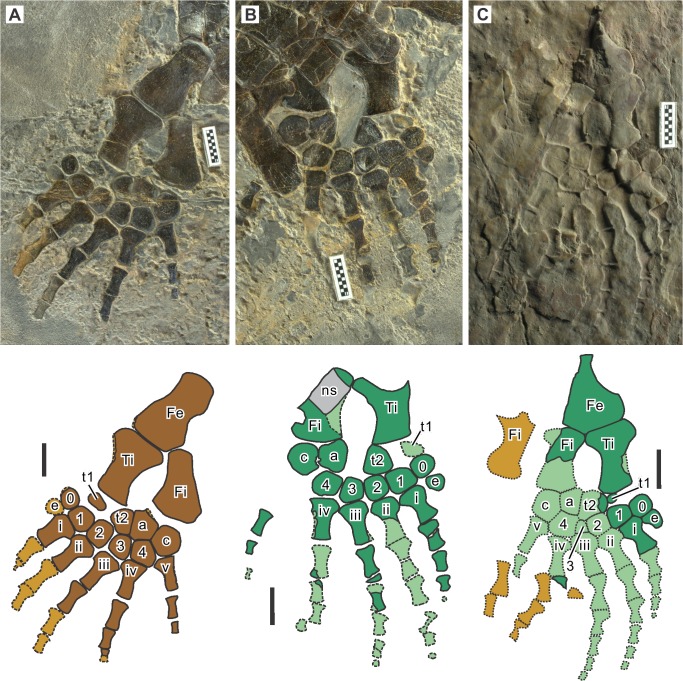
Hindlimb of *Eretmorhipis carrolldongi* gen. et sp. nov. **A, WGSC V26020, left; B, WGSC V26020, right; C, IVPP V4070.** Symbols: e, extra preaxial metatarsal; a, astragalus; c, calcaneum; Fe, Femur; Fi, fibula; ns, neural spine; t1, preaxial extra proximal tarsal; t2, postaxial extra proximal tarsal; Ti, tibia; 0–4, distal tarsals; i-v, metatarsals. Colors as in Fig 5. Scale bars are 1 cm long.

WGSC 26020 (Figs 2A, 3, 4, 5A, and 6AB), an almost complete postcranial skeleton preserving from the posterior cervical region to the tip of the tail. The trunk is exposed dorsally, while the pelvic region and the tail in left lateral view.

#### Referred Specimen

IVPP V4070 (Figs 2B, 5B and 6C), a skeletal impression spanning from posterior cervical to posterior caudal vertebrae. The impressions are not very well defined overall, except those of some limb bones. Parts of the right forelimb and hindlimb are preserved as bones. The individual is exposed in right lateral view, although impressions reveal the shape of the other side.

#### Diagnosis

(Autapomorphies) radiating manual and pedal digits forming fan shaped autopodium; manual digits short, with maximum of four phalanges; humeral axis tilted relative to forearm axis; olecranon process of ulna modestly developed; sesamoid in proximal carpal row; extra proximal carpal proximal margin concave; preaxial extra proximal tarsal small; about 30 dorsal vertebrae; dermal armor third layer elements large, spanning maximum of four vertebral segments; second layer dermal elements free of overlying third layer elements present; two caudal vertebrae with both hemal spine and caudal ribs; lateral gastral elements large.

#### Locality and Horizon

WGSC V26020 is from the Uppermost Spathian (Lower Triassic) Jialingjiang Formation[13], exposed in Yingzhishan, Yuan'an County, Hubei Province, China. IVPP V4070 was reported to have been from the same geologic formation exposed near Tuling, Baihechuan, Xunjian District, Nanzhang County, Hubei Province, China [2, 6].

### Description

The holotype is slightly larger than the referred specimen, by about 25% on average (Table 1). The two specimens closely resemble each other yet have several differences apart from size, as described below. Both specimens lack the skull, so comparisons will be limited to the postcranium (Fig 2). The description below will be based on WGSC V26020, unless otherwise noted.

**Table 1:** Selected measurements of the two specimens in mm.

		WGSC V26020	IVPP V4070
Dorsal	Length	318	255
Humerus	Length	41.91	32.29
	Proximal Width	22.25	—
	Distal Width	28.86	—
Radius	Length	34.31	25.20
	Proximal Width	14.50	14.34
	Distal Width	17.82	13.61
Ulna	Length	34.36	26.04
	Proximal Width	14.52	—
	Distal Width	16.82	15.73
Femur	Length	26.30	—
	Proximal Width	11.22	—
	Distal Width	16.21	13.51
Tibia	Length	20.24	16.92
	Proximal Width	16.72	12.20
	Distal Width	9.64	—
Fibula	Length	21.60	16.74
	Proximal Width	7.37	—
	Distal Width	15.27	9.03

#### Axial Skeleton

The holotype is estimated to have 30 dorsal, 2 sacral, and 56 caudal vertebrae. The dorsal count assumes that the three most craniad dorsals are not preserved based on the width of the gap, which has some impressions. The beginning of the dorsal series was estimated to start from the impression of the proximal end of the first elongated rib on the left side. Seven more were estimated to be missing from the damaged posterior dorsal region based on the width of the damaged area. Hereafter, dorsal vertebrae will be numbered based on the assumptions stated above.

The sacral vertebrae were identified based on their extensive rib facets that span almost the entire height of the vertebral centra, comprising both the diapophysis and parapophysis (Fig 4). The location of the left ilium suggests the same identification. The ilium appears to have moved minimally during deposition because the left half of the pelvic girdle and femur are still in articulation. The rate of shrinking of lateral gastral elements also suggests that the pelvic girdle is still near its original position. The referred specimen is estimated to have about 30 dorsal vertebrae based on impressions. There may be errors in these values because vertebral numbers were in part calculated from distances when vertebral boundaries were not well-defined. The dorsal counts are higher than in *Hupehsuchus nanchangensis*, which usually has 28 dorsals as in its holotype, but much lower than in *P*. *longus*, with 38. The caudal count is also higher than in *H*. *nanchangensis*, which has about 10 vertebrae less than the new species—the caudal count of *P*. *longus* is unknown. Cervical vertebrae are poorly known, based only on weathered bones in the referred specimen. It is difficult to establish a reasonable cervical count.

Neural spines are low, as observed by [2]. As with *Nanchangosaurus suni* [7], neural arches are about the same height throughout the trunk region, while the neural spines change their heights depending on the position. Thus, neural spines are lower than the neural arches anteriorly in the trunk, and become about as high as the latter near the tenth dorsal vertebra. In the sacral region, where the neural spines are the highest within the body, they are about twice as high as the neural arches. Rudimentary transverse processes of the neural arch may be recognized near the eighth dorsal vertebra (Fig 4A) but they seem to be absent in the mid-dorsal region, near the 19th dorsal vertebra (Fig 4B).

At least some neural spines are divided into two segments, as in other hupehsuchians, although the preservation does not allow us to clarify the exact distribution of this feature along the vertebral column. It is present as posteriorly as the first caudal vertebra (Fig 4), whereas its anterior extent is uncertain. The second segment is usually tightly fused with the first-layer dermal armor elements that will be described later. It is also fused with the first segment of the neural spine in many of the vertebrae, obscuring the boundary as seen in Fig 3. Additionally, in most hupehsuchians, the base of the second neural-spine segment is narrower than the first segment, allowing the identification of the boundary between the two segments. Such a constriction, however, is nearly absent in the craniad half of the trunk in *Eretmorhipis carrolldongi*, further obscuring the boundary. The articulation between the two neural spine segments loosens toward the pelvic girdle, as is shown in Fig 4.

The first complete hemal spine is associated with the fifth caudal vertebra. However, the fourth caudal has a facet for a hemal arch, as well as associated fragments (Fig 4). The third caudal lacks such an articular facet, so the first three caudal vertebrae lack the hemal spines. The impression of the first hemal spine, which is half damaged, suggest that it was short and strongly curved, unlike the more posterior hemal spines (Fig 4). Anterior hemal spines are nearly vertical, being tilted about 75° from the tail axis. They quickly become more horizontal within the first seven vertebral segments, so that the seventh hemal spine, associated with the 10th caudal vertebra, is tilted only about 20° from the tail axis. This angle stays almost constant throughout the remaining ~50 vertebrae. These more horizontal hemal arches touch each other and span two to three vertebral segments, likely limiting the flexibility of the tail. They are broad and robust in the holotype, contacting each other, but appear more slender in the referred specimen, where there are gaps between adjacent hemal spines. The hemal spine morphology in the referred specimen is closer to that of *Hupehsuchus nanchangensis* than of the holotype.

Ribs exhibit a mixture of features of *Hupehsuchus nanchangensis* and *Parahupehsuchus longus*. The first few ribs behind the shoulder girdle are similar to those of *P*. *longus*, in that they have extensive posterior flanges that extend distally, making the ribs appear parallel, not tapered (Fig 3A). A small anterior flange also seems to exist at least in some ribs in the area. More caudad ribs, however, have posterior flanges that do not extend as far distally (Fig 3B), giving each rib a clearly tapered appearance as in *H*. *nanchangensis*. Given that the most-caudad dorsal ribs of *P*. *longus* also exhibit tapering, the condition in the new species may be interpreted as transitional between those of *H*. *nanchangensis* and *P*. *longus*. Both types of ribs are pachyostotic, without longitudinal grooves seen in *Nanchangosaurus suni* and *Eohupehsuchus brevicollis* [4]. Each dorsal rib overlies the craniad rib.

Rib articulation resembles that seen in *Parahupehsuchus longus* in the anterior dorsal region. As best seen in the 10th and 11th dorsal vertebrae, the holocephalous rib heads (Fig 3, light blue) are much wider than the synapophysis composed of the diapophysis (light brown) and parapophysis (yellow). This is because of the presence of anterior and posterior flanges. The 10th dorsal rib is broken proximally, revealing the articular facet beneath (mint green in Fig 3A). This corresponds to the dorso-craniad extension of the parapophysis that provides a second articulation for the rib in *P*. *longus*. Thus, at least the anterior dorsal ribs have a double articulation in *Eretmorhipis carrolldongi*, forming a short structure that resembles the bony body tube in *P*. *longus*. In the posterior dorsal region, however, the rib heads are as wide as the corresponding synapophysis, as seen in the 18th and 19th dorsal ribs (Fig 3B). There does not seem to be the dorso-craniad extension of the parapophysis that forms a secondary articulation between the ribs and vertebrae. Note that such a double articulation is also present in some ribs of *Hupehsuchus nanchangensis*; although previously unrecognized [3], this is now reflected in the data matrix.

There is a short rib that underlies the left ilium (Fig 4), which is identified as a sacral rib. Otherwise, sacral ribs are poorly known. Caudal ribs are only known as fragments, but the articular facets on the left side of the caudal vertebral centra (Fig 4) suggest that there were five pairs of caudal ribs. This number is higher than in any other hupehsuchian for which caudal rib facets are preserved. The fourth and fifth caudal vertebrae therefore have both the caudal rib and hemal arch. At most one caudal vertebra has both elements in other hupehsuchians.

Gastral elements are well-developed. They are best exposed on the left side of the body, where ribs curve caudally to reveal the structures beneath them (Fig 2). The lateral gastral elements are boomerang-shaped as in other hupehsuchians. They are highly asymmetric, with a short medial process and a long lateral process, as in *Parahupehsuchus longus*. The lateral elements are long, rivaling the distance covered by four or more vertebral segments nearby. One-to-one correspondence between ribs and gastral elements is not present; gastral elements are more abundant than ribs. In the mid-dorsal region, there are about1.5 lateral gastral elements per rib. The gastral elements are more abundant in *P*. *longus*, *Nanchangosaurus suni*, and *Eohupehsuchus brevicollis*, which have about two elements per rib. Therefore, *Eretmorhipis carrolldongi* is unique for having less but larger gastral elements than other hupehsuchians. Median elements are poorly known, except the ones exposed craniad of the pelvic girdle (Fig 4). They are round in cross section, unlike in basal hupehsuchians.

There are three layers of dermal ossicles above the neural spines as in most other hupehsuchians except *Nanchangosaurus suni* (Fig 4), but their arrangement is unique in *Eretmorhipis carrolldongi*. The third layer elements are unusually large, and increasingly become larger and sparser toward the pelvic region, posterior to which they grow smaller again. At maximum, they are slightly longer than three vertebral segments, and span up to four vertebral segments depending on their position. In other hupehsuchians, third-layer elements are much shorter: they are only about one vertebral-segment long in *Eohupehsuchus brevicollis* and 1.5 to 2 times in *Hupehsuchus nanchangensis* and *Parahupehsuchus longus*. In the mid-trunk region of *Er*. *carrolldongi*, there seems to be one third-layer element per every five second-layer elements (Fig 3C). Thus, there is a gap between adjacent third-layer elements, and the second-layer element in the gap is enlarged (enl. in Fig 3C) unlike other second-layer elements, which usually appear ‘squished’ below the large third-layer element. Such second-layer elements that are completely free of third-layer elements are absent from other hupehsuchians. The dermal armor is already present in the anterior dorsal region; their presence in the cervical region cannot be confirmed because of the poor preservation in the area. Posteriorly, the last third-layer element is associated with the 12th and 13th caudal vertebra, and the last first-layer element is located atop the second caudal neural spine. The last second-layer element is found above the sacral neural spines. Therefore, the third-layer elements persist without the first two layers lying below them in the caudal region. The presence of dermal armor in the anterior caudal region is shared with *P*. *longus* and *Eo*. *brevicollis*.

#### Appendicular skeleton

At least some elements are known only from natural molds in every limb. These molds, however, are usually well-defined, allowing us to draw unambiguous bone outlines in many cases (Figs 5 and 6). The pectoral girdle of *Eretmorhipis carrolldongi* is poorly exposed in either specimen, so a detailed description cannot be given at this point. Limb bones tend to be more robust in the smaller specimen (Table 1).

The humerus has an anterior flange that is not very extensive. The preaxial margin of the flange is almost straight. The bone is unique among hupehsuchians in that its longitudinal axis is inclined relative to the general direction of the more distal parts of the limb, i.e., the humeral head is pointing somewhat postaxially. The radius is a robust bone that is almost rectangular, with a slight constriction of the shaft. Its preaxial margin is thin mid-shaft, probably representing a small anterior flange. However, surface striations are too poorly preserved to firmly establish the presence of a flange. The ulna is slightly narrower than the radius. Its proximal end expands postaxially to form a structure that may be referred to as a small olecranon. However, the homology of this structure is uncertain. The distal end of ulna is slightly expanded, as in other hupehsuchians and basal ichthyopterygians. The stylopodial and zeugopodial elements are sub-equal in length, while the manus is marginally shorter than the previous two combined.

There are four proximal carpals, as in *Parahupehsuchus longus*. The extra element between the radius and intermedium is most likely a neomorph [3], although a possibility remains that this element is indeed a dislocated centrale, as previously suggested [2]. This neomorph is concave proximally in both specimens, unlike in *P*. *longus* [3] or the specimen figured in fig. S1A of [8]. There is a sesamoid in the proximal carpal row in both specimens. However, the location of the sesamoid differs between the two (Fig 5). The one in the holotype (Fig 5A) may be interpreted as the pisiform given its position, but the element is absent in the referred specimen (Fig 5B), which instead has a similarly sized element preaxially. There is a tendency in Parahupehsuchinae to have neomorphs in the proximal carpal and tarsal rows [3], so these elements that are inconsistently present may also be neomorphs. The pisiform is unknown among hupehsuchians. Distal carpal 5 is absent, as in other ichthyosauromorphs. There is an extra distal carpal preaxially (0 in Fig 5), which is followed distally by an extra preaxial metacarpal (e in Fig 5). However, there is no phalanx in this extra preaxial digit, unlike in *P*. *longus*. The fifth metacarpal appears slightly hooked in the holotype but not in the referred specimen.

The preserved phalangeal formula for the manus is 0-4-3?-4-3-2 in WGSC V26020 (Fig 5), although one phalanx is probably missing from digit 2 because its last preserved phalanx is still large, unlike in digits 1 and 3. Note that the formula starts from digit 0 (extra preaxial digit) to facilitate comparisons with polydactylous species. The forelimbs of IVPP V4070 are more incomplete than in WGSC V26020, and the observed phalangeal formula corresponds to the one given above. This formula is modest compared to what is known for the type specimens of *Parahupehsuchus longus* (1-5-5-3?-1?-1), or *Hupehsuchus nanchangensis* (0-5-5-5-4-2). Therefore, the manus of *Eretmorhipis carrolldongi* is shorter and wider than that of *P*. *longus* and *H*. *nanchangensis*. There is a tendency in Hupehsuchia for digits 1 to 3 of the manus to share the maximum phalangeal count. This feature seems also to be present in *Eretmorhipis carrolldongi*.

The pelvic girdle resembles that of other hupehsuchians. As mentioned earlier, the left half of the girdle and the left femur seem to be preserved in articulation. The ilium is a short and robust rod with expanded extremities for rib articulation distally, and participation in the formation of the acetabulum proximally (Fig 4). It is thicker proximally than distally. Its distal end is sufficiently wide to accept two sacral ribs but probably not three, unless the third is narrow. The pubis is a round plate. There is a notch posteriorly, probably representing an open obturator foramen. The ischium is incompletely known. It appears to be a plate-like bone that may be smaller than the pubis.

As with the humerus, the femoral axis is slightly inclined relative to the longitudinal axis of the more distal part of the limb, so that the femoral head points somewhat postaxially (Fig 6A). The distal end of the femur is mostly occupied by the tibial facet, with a small fibular facet deflected postaxially. There is no flange preaxially or postaxially in the femur, unlike in the humerus. The tibia and fibula are more robust than the radius and ulna, respectively.

There are four proximal tarsals, of which two preaxial ones are identified as neomorphs, as in *Parahupehsuchus longus* [3]. The more preaxial neomorph (t1 in Fig 6) is smaller than the postaxial counterpart (t2) in both specimens. This is in contrast to the condition in *P*. *longus*, where t2 is rudimentary and t1 robust [3]. There are five distal tarsals including an extra element preaxially (0 in Fig 6). There are six metatarsals, again including an extra preaxial element (e in Fig 6). The extra preaxial digit, however, lacks any phalanx.

The phalangeal formula for the pes is 0-4-5-5-4-3 in WGSC 26020 (Fig 6B), and 0-4-5-5-?-? in IVPP V4070 (Fig 6C). Therefore, the pes has more phalanges than the manus in *Eretmorhipis carrolldongi*. The fifth digit is noticeably narrower than the rest of the digits, unlike in the manus.

## Discussion

The two specimens of *Eretmorhipis carrolldongi* are not identical in every feature. Most notably, the shape of hemal spines differ significantly between the two, and each limb bone is more robust in IVPP V4070 than in WGSC V26020. We still referred IVPP V4070 to *Er*. *carrolldongi* based on its overall morphological similarities with the holotype that are described above, including the limb bone shape and arrangements, dorsal vertebral count, and relative neural spine heights. A species is expected to have morphological variations resulting from many reasons, such as the gender, ontogeny, genetic variations, and individual history. Given that the degree of these variations is poorly understood for *Er*. *carrolldongi*, it seems plausible to stay conservative and consider the two specimens to be conspecific.

We did not consider the new species to be congeneric with *Parahupehsuchus longus* because there is a substantial list of differences between the two, as evident from the diagnosis of the new species. For example, the dorsal vertebral count of *P*. *longus* (38) is drastically different from that of other hupehsuchians (26–30), and its body trunk forms a long bony body tube. *Eretmorhipis carrolldongi* has a partial body tube as described above but it is too short to be equated with that of *P*. *longus*. Also, *P*. *longus* is unique among hupehsuchians for having the most flipper-shaped forelimb with a pointed tip as in some dolphins, while *Er*. *carrolldongi* is also unique in the other direction, for having a completely radiating set of digits unlike in other hupehsuchians. We found it inappropriate to place all those extreme variations within a single genus, when other genera are more similar to each other.

We did not refer the specimen described by [8] to *Eretmorhipis carrolldongi*. The specimen differs from *Er*. *carrolldongi* in several features. The specimen has a forelimb that is about the same size as that of the holotype of *Er*. *carrolldongi*, yet its carpals are not as well-ossified as in the latter, suggesting that the specimen is less mature than the latter. However, the specimen has two extra preaxial digits down to phalanges, as opposed to one extra digit without a phalanx in *Er*. *carrolldongi*. Also, it has five phalanges in the longest digit, as opposed to four in the new species. It is very unlikely that the specimen is a juvenile of *Er*. *carrolldongi*, or vice versa. Further discussion would require a full description of the specimen.

Hupehsuchian forelimbs exhibit a variety of morphological adaptations while sharing a basic design. One extreme is represented by *Parahupehsuchus longus*, which has a pointed forelimb [3] that is best described as a flipper [14]. The other extreme is seen in *Eretmorhipis carrolldongi*, whose forelimb forms a paddle ending with a fan-shaped expansion. The forelimb of *Hupehsuchus nanchangensis* is intermediate between the two, for being a paddle with a modest distal expansion [2, 3, 6]. The presence of such a variation is in contrast to what is known for ichthyopterygians, whose forelimbs are all flipper-shaped without an exception despite their much higher taxonomic diversity [15, 16]. The variation in forelimb shape suggests that hupehsuchians had species-dependent behavioral adaptations, probably to different types of habitats. Moreover, the hind limb exhibits a different pattern than the forelimb. *Parahupehsuchus longus* and *Hupehsuchus nanchangensis* share a similar skeletal outline of the hind limb, which is a pointed flipper. *Eretmorhipis carrolldongi*, however, has a distally fanning paddle. The combination of fore- and hind limbs further enhances morphological, and hence behavioral, variations among hupehsuchians. Such adaptations likely enabled hupehsuchians to divide resources, which allowed them, in turn, to have a high taxonomic diversity within a limited geographic area.

The partial body tube of *Eretmorhipis carrolldongi* is an evolutionary enigma. It is morphologically intermediate between the trunks of *Hupehsuchus nanchangensis* and *Parahupehsuchus longus*; the former has a heavily ossified trunk with some inter-costal space while the latter has a rigid body tube without any space between bones [3]. The limited body tube of *Er*. *carrolldongi*, which occupied only about a craniad third of the trunk, provided a rigid chest region while leaving some flexibility to the rest of the trunk. The body tube in Parahupehsuchinae may have first evolved as an adaptation that rigidified the pectoral region—such a rigid chest would allow effective paddling or rowing using a pair of large paddles, as in turtles and basal sauropterygians. If so, the extensive body tube of *P*. *longus* may have evolved from such a small body tube into an enhanced protective device (i.e., hupehsuchian trunk is already heavily built even without a body tube), while its original function became not as important as the forelimbs became small. While such an interpretation may appear reasonable, it is equally possible at this point that the extensive body tube of *P*. *longus* is primitive for the Parahupehsuchinae, and the shortened version in *Er*. *carrolldongi* is derived. A further discovery of an intermediate species is necessary to test alternative interpretations.

## Supporting Information

S1 File Data matrix for phylogenetic analysis. (DOCX)
