# Supplementary material for: A New Specimen of Carroll’s Mystery Hupehsuchian from the Lower Triassic of China
Source: PLoS One. 2015 May 27;10(5):e0126024. doi: 10.1371/journal.pone.0126024 (PMC4446317; doi:10.1371/journal.pone.0126024)
Supplement: S1 File — (DOCX) [file pone.0126024.s001.docx]

**S1 File. Data matrix for phylogenetic analysis**

**Character Matrix**

Hovasaurus 00000000000000000000000000?0000?0?000

Cartorhynchus 0101101100??0001000000?010?0000?0?000

Chaohusaurus 0111000100000(0 1)010000100010?1000?0?000

Nanchangosaurus 10(0 1)010110??????????101100101011010100

Eohupehsuchus 1010100110?10010?0?001101101011010111

Hupehsuchus 10(0 1)01?0110?1(0 1)010100101111100011111110

Parahupehsuchus ?????00111011110111?11111110111111111

WGSC.V26020 ?????10011111100101?1111?110?11111111

IVPP.V4070 ????????11111100101?111??1?01?????1?1

WuEA ?????10011001100111?1????????????????

**Character Description**

(1) Snout, flattened: (0) FALSE; (1) TRUE.

(2) Nasal extended rostrally beyond external naris: (0) FALSE; (1) TRUE.

(3) Frontal, participation in orbital margin: (0) present; (1) absent.

(4) Large scleral ring filling the orbit: (0) absent; (1) present.

(5) Teeth: (0) small; (1) edentulous.

(6) Humerus, anterior margin: (0) concave; (1) straight or convex.

(7) Manual zeugopodials shortened: (0) FALSE; (1) TRUE.

(8) Ulna, small olecranon extending posteriorly beyond humerus: (0) present; (1) absent.

(9) Carpal development: (0) delayed; (1) normal.

(10) Extra proximal carpal: (0) absent; (1) present.

(11) Manual lateral centrale, proximal margin: (0) convex; (1) concave.

(12) Radiale larger than other proximal carpals: (0) FALSE; (1) TRUE.

(13) Extra ant. distal carpal: (0) absent; (1) present.

(14) Extra anterior metapodial: (0) absent; (1) present.

(15) Digits 1-3 'bundled': (0) FALSE; (1) TRUE.

(16) Digital separation: (0) present at least partly; (1) absent.

(17) Manual digit 1 hyperphalangeal and with max number of phalanges: (0) FALSE; (1) TRUE.

(18) First phalanx in manual extra anterior digit: (0) absent; (1) present.

(19) Extra proximal tarsal: (0) absent; (1) present.

(20) Cervical count: (0) 6 or less; (1) 9 or more.

(21) Dorsal count: (0) 29 or less; (1) 30 or more.

(22) Anterior dorsal neural spine, second segment: (0) absent; (1) present.

(23) Posterior dorsal neural spine, first segment, interspinal space: (0) present; (1) absent.

(24) Dorsal rib articulating with two vertebrae: (0) absent; (1) present.

(25) Parapophysis elevated above posterior dorsal centra margin: (0) absent; (1) present.

(26) Rib posterior flange: (0) absent; (1) present.

(27) Anterior dorsal rib, posterior flange extent: (0) only proximally; (1) extensive.

(28) Rib, pachyostosis midshaft: (0) present; (1) absent.

(29) Ribcage, depth: (0) swollen midway; (1) semi-constant.

(30) Gastralia, anterior flange overlapping anterior gastralia: (0) absent; (1) present.

(31) Lateral gastralia boomerang-shaped, pointing anteriorly: (0) absent; (1) present.

(32) Lateral gastral element each approximately symmetrical: (0) TRUE; (1) FALSE.

(33) Median gastralia v-shaped, pointing posteriorly: (0) absent; (1) present.

(34) Median gastralia cross-section: (0) flattened; (1) round.

(35) Dermal armor above dorsal neural spine, first layers: (0) absent; (1) present.

(36) Dermal armor above dorsal neural spine, third layer: (0) absent; (1) present.

(37) Dermal armor above caudal vertebrae with haemal arches: (0) absent; (1) present.

**Character correspondence**

| This paper | Ref. 4 | Ref. 3 |
| --- | --- | --- |
| 1 | 1 | 1 |
| 2 | 2 | 2. mod |
| 3 | 3 | — |
| 4 | 4 | — |
| 5 | 5 | 3 |
| 6 | — | — |
| 7 | 6 | — |
| 8 | — | — |
| 9 | 7 | — |
| 10 | 8 | 8. mod |
| 11 | — | — |
| 12 | 9 | 9 |
| 13 | 10 | 10 |
| 14 | 11 | 11 |
| 15 | 12 | 12 |
| 16 | 13 | — |
| 17 | 14 | 13 |
| 18 | — | — |
| 19 | 15 | — |
| 20 | 16 | 14.mod |
| 21 | 17 | 14.mod |
| 22 | 18 | 15 |
| 23 | 19 | 16 |
| 24 | 20 | — |
| 25 | 21 | — |
| 26 | 22 | 17 |
| 27 | — | — |
| 28 | 23 | — |
| 29 | 24 | 18 |
| 30 | 25 | 19 |
| 31 | 26 | 20 |
| 32 | 27 | — |
| 33 | 28 | 21 |
| 34 | 29 | — |
| 35 | 30 | 23 |
| 36 | 31 | 24 |
| 37 | 32 | 25 |
| — | — | 4 |
| — | — | 5 |
| — | — | 6 |
| — | — | 7 |
| — | — | 22 |
